# Relationship between self-disclosure and anticipatory grief in patients with advanced lung cancer: the mediation role of illness uncertainty

**DOI:** 10.3389/fpsyg.2023.1266818

**Published:** 2023-12-08

**Authors:** Nan Zhang, Han Li, Huaxin Kang, Yinglan Wang, Zhitong Zuo

**Affiliations:** ^1^Wuxi School of Medicine, Jiangnan University, Wuxi, China; ^2^Department of Respiratory Medicine, Affiliated Hospital of Jiangnan University, Wuxi, China

**Keywords:** lung cancer, anticipatory grief, illness uncertainty, self-disclosure, structural equation model

## Abstract

**Objective:**

To study the relationship between self-disclosure, illness uncertainty (IU) and anticipatory grief (AG) in patients with advanced lung cancer.

**Methods:**

This is a cross-sectional study using convenience sampling method, in which 316 patients with advanced lung cancer who were hospitalized in a tertiary hospital in Wuxi City, China, from November 2022 to April 2023 were sampled. The Preparatory Grief in Advanced Cancer Patients, Mishel Uncertainty in Illness Scale, and the Distress Disclosure Index Scale (DDI) were selected to analyse the status quo, correlations, and the mediating effect of illness uncertainty on the relationship between self-disclosure and anticipatory grief in advanced lung cancer patients.

**Results:**

The total self-disclosure score of advanced lung cancer patients was (36.35 ± 9.25), the total score of IU was (56.92 ± 15.65), and the score of AG was (52.29 ± 9.08); the results of correlation analyses showed that IU was negatively correlated with self-disclosure in advanced lung cancer patients (*p* < 0.05) and positively correlated with AG (*p* < 0.05), and self-disclosure was negatively correlated with AG (*p* < 0.05);the mediating effect rate of IU between self-disclosure and AG in advanced lung cancer patients was 49%.

**Conclusion:**

The AG of advanced lung cancer patients was at a medium-high level, and IU had a significant mediating effect between self-disclosure and AG of advanced lung cancer patients; by increasing the level of patients’ self-disclosure, IU could be effectively alleviated, and ultimately the AG of the patients could be reduced.

## Introduction

According to cancer statistics released in the United States, lung cancer has become the second most prevalent cancer, but it still occupies the top spot for cancer deaths over 50 years of age (Siegel et al., 2023). The World Health Organisation’s International Agency for Research on Cancer (IARC) recently released the latest global cancer burden data for 2020, and China ranked first in the world in terms of the number of new cases and deaths, with the number of new cancers reaching as high as 4.57 million, accounting for 23.7 per cent of the total global cancer mortality rate (Sung et al., 2021). In China, the incidence rate of lung cancer ranks first and far ahead, accounting for 20 percent of China’s cancer incidence rate; however, nearly 70 percent of lung cancer patients in China are diagnosed at an advanced stage at the beginning of the diagnostic process, and the five-year survival rate of advanced-stage patients is less than 5 percent, resulting in a lung cancer mortality rate of 27 per cent of the total mortality rate of cancers (Luo et al., 2021). Patients with advanced lung cancer often lose the opportunity for surgical treatment, and are mostly treated with chemotherapy, radiotherapy and immunotherapy, which requires patients to undergo enormous economic pressure while also facing changes or loss of physical and social functions and various uncertainties, with significant psychological problems and widespread anticipatory grief (Li et al., 2022).

More and more scholars are now focusing on this aspect of advanced cancer, anticipatory grief is the patient’s emotional response to losses experienced in the past, those that are occurring and those that will arise in the future (Tsilika et al., 2009). Anticipatory grief occurs on three psychological, interpersonal, and sociocultural levels and is a multidimensional experience (Coelho and Barbosa, 2017). Anticipatory grief is a psychological process experienced by every patient with advanced cancer (Khanipour-Kencha et al., 2022). As the disease progresses, loss of function and potential loss of life accumulate, anticipatory grief is triggered and occurs and develops in a wave-like trajectory, which can be alleviated by psychological support and counselling, and medication is often ineffective and even has some side effects (Periyakoil and Hallenbeck, 2002). AG can have a positive correlation with negative emotions such as depression and can have a mutually reinforcing effect, aggravating the patient’s psychological distress. Anticipatory grief and depression share many similarities in clinical symptoms, such as sleep disturbance, loss of appetite, and weight loss. The difference between the two is that persistent low mood or irritability pervades all aspects of the patient’s life, which is characteristic of depression, with disorders of self-image as well as recurring feelings of worthlessness and suicidal tendencies, and symptomatic relief requires the use of antidepressant drugs and psychological interventions at the same time; when anticipatory grief occurs, self-esteem is not impaired, and it is mainly accompanied by waves of grief associated with loss, which does not require medication, and can be alleviated by psychological interventions (Periyakoil and Hallenbeck, 2002). Grief and depression, as distinct but related processes, can lead to intense suffering (Periyakoil and Hallenbeck, 2002).

Anticipatory grief is a natural process that needs to be acknowledged, accepted and expressed by the patient, and the presence of loss early in the disease is an opportunity to encourage the patient to express their grief so that they can be better prepared when faced with some of the ensuing loss series (Barber, 2022). Self-disclosure is the process by which patients share their feelings, emotions, and experiences with others. Positive self-disclosure can promote patients’ adjustment and adaptation to stressors, alleviate cancer patients’ negative psychological experiences, and reduce the level of negative emotions (Sajjadi et al., 2016; Davis et al., 2017). However, there is no scholarly research on the relationship between self-disclosure and anticipatory grief, which may lead to a lack of direction and evidence for anticipatory grief intervention plans. Therefore, based on the current state of research, we believe it is necessary to investigate the mechanism of the relationship between self-disclosure and anticipatory grief.

Illness uncertainty, which refers to an individual’s lack of ability to identify things related to an illness, has been revealed to be prevalent in up to 60% of cancer patients (Groenvold et al., 2006). As patients face the possibility of death at advanced diagnosis, severe unexplained symptoms and uncertainty about treatment elicit negative emotions such as fear, anxiety, and grief (Cheng et al., 2010). Results of qualitative studies of advanced cancer patients with high levels of anticipatory grief show that the main and prevalent emotional appeal is uncertainty about the disease (Liu and He, 2017), but the mechanisms by which illness uncertainty affects anticipatory grief are not clear. Based on a survey of the literature, lack of emotional disclosure may prevent others from identifying patients’ needs for emotional support and disease-related information, leading to psychological dysregulation. Therefore, we hypothesized that self-disclosure may modify the effect on anticipatory grief by putting low IU in patients with advanced lung cancer.

**Figure 1:**
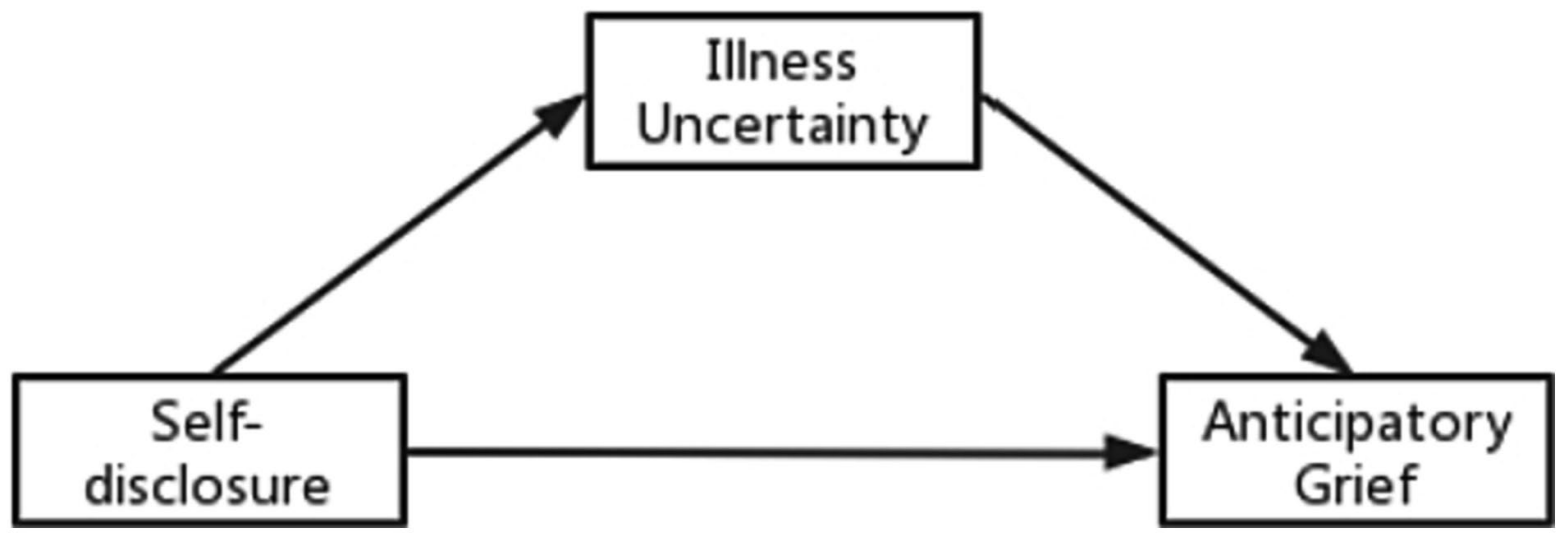
The hypothesized model.

Therefore, the main aim of this study was to investigate the relationship between self-disclosure, illness uncertainty (IU) and anticipatory grief (AG) in patients with advanced lung cancer. We made the following hypotheses: 1. Lower AG was associated with higher self-disclosure. 2. IU mediated self-disclosure and AG in patients with advanced lung cancer. Figure 1 shows the hypothetical model.

## Materials and methods

### Participants

This is a cross-sectional study using convenience sampling method. Patients with advanced lung cancer admitted to the Department of Respiratory Medicine and Department of Oncology of a tertiary hospital in Wuxi, China, from November 2022 to May 2023 were the study subjects. Inclusion criteria: (1) patients with pathological diagnosis of advanced lung cancer (stage III or IV); (2) patients aged >18 years; (3) able to communicate and cooperate with the study normally in Chinese; and (4) patients gave informed consent and participated voluntarily. Exclusion criteria: (1) non-primary lung cancer or other malignant tumors; (2) history of psychiatric illness or taking psychiatric drugs; (3) inability to effectively communicate and understand the questionnaire content. In structural equation modelling (SEM) analyses, the ratio of sample size to the number of observed variables was at least between 10:1 and 15:1. In this study, we invited 323 subjects who met the exclusion criteria, among which 3 patients were unable to participate due to disease exacerbation; Three patients refused outright; One patient was not interested. Therefore, our final sample included 316 patients, with a total response rate of 97.9%, which met the requirements of SEM analysis.

### Procedure

The Chinese version of the questionnaire was used to collect information from participants in the form of patient self-report. The questionnaire consisted of the following four main parts: sociodemographic as well as disease-related characteristics, the Distress Disclosure Index (DDI), the Mishel Uncertainty about Illness Scale (MUIS), and the Preparatory Grief in Advanced Cancer Patients (PGAC). Relevant data and information were collected in the form of an on-site survey, and the purpose and significance of this survey were explained to patients and their families, as well as to help participants who had difficulty reading the questionnaire. Uniform training was provided to the investigators before the start of the study, and the investigators were required to acquaint themselves with the use of the various questionnaires and scales, to answer the questions on the spot using uniform instructions, and to retrieve the questionnaires on the spot after they were completed.

### Ethics approval

The study was approved by the Ethics Committee of JNU. In accordance with the Declaration of Helsinki, the researchers explained the purpose and process of the study and informed patients that they could withdraw at any time, all patients provided written informed consent, and all data were anonymised to ensure participant privacy.

### Measures

### Sociodemographic

Includes socio-demographic data (age, sex, marital status, education, monthly household income, main carer, and character) and disease-related data (time of diagnosis of the disease, number of symptoms of discomfort).

### Self-disclosure

The degree of self-disclosure was measured by the Distress Disclosure Index (DDI), and this study used the Chinese version of the DDI (Xinmin, 2009). The questionnaire consists of 12 items, and is rated on a 5-point Likert scale, with positive items scored 1–5, and negative items scored 5–1, and the total score of the scale ranges from 12 to 60 points, divided into three levels, with 12–29 points being a low level, 30–44 points being a medium level, and 45–60 points being a high level. 30–44 as medium level and 45–60 as high level, with higher scores indicating greater likelihood of emotional expression. In this study, the Cronbach’s alpha coefficient for the DDI in patients with advanced lung cancer was 0.798.

### Illness uncertainty

Mishel Uncertainty in Illness Scale (MUIS) was first developed by Mishel in 1981 and has since been revised and widely used in clinical practice (Mishel, 1981). In this study, the Chinese version of MUIS for Chinese patients with malignant tumors was used (Zengjie et al., 2018). The scale is divided into three dimensions, namely, ambiguity, lack of clarity and unpredictable, with a total of 20 items. Each item was scored from 1 to 5 points from “strongly disagree” to “strongly agree,” with a total score of 20 to 100 points. The higher the score, the higher the level of illness uncertainty of patients. The Cronbach’s α coefficient of the Chinese version of MUIS was 0.825.

### Anticipatory grief

Preparatory Grief in Advanced Cancer Patients Scale was developed by Mystakidou et al. (2005) to assess the level of anticipatory grief in patients with advanced cancer. The scale has 5 dimensions and 26 items, containing somatic symptoms (4 items), sad and angry emotions (12 items) perceived social support (3 items), attitudes towards death (4 items) and spiritual needs (3 items) (Ying et al., 2018). The Likert 4-level scoring method was adopted, with 0 indicating “disagree,” 1 indicating “somewhat disagree,” 2 indicating “somewhat agree,” and 3 indicating “agree.” The total score ranges from 0 to 78. The higher the score, the higher the level of presentiment grief. In this study, the Cronbach’s a coefficient of the scale was 0.891.

### Statistical analysis

Statistical analyses were performed using IBM SPSS Statistics 26.0 (IBM Corporation, United States). Comparisons of differences between multiple groups of normally distributed measures were performed using single-factor ANOVA, and Pearson correlation analyses were used to explore the correlation between each normally distributed measure. The mediation effect test was analysed using Process V4.1 protocol and re-validated using AMOS, a validated statistical analysis software. Bootstrap method was used to test the mediation effect, and the bootstrap 95% CI of the mediation effect did not contain 0, which indicated that the mediation effect was significant, and the *p* < 0.05 was considered to be statistically significant (Hayes, 2009).

## Results

### Sociodemographic and psychosocial characteristics

A total of 316 patients with advanced lung cancer participated in this study, and the descriptive statistics of all study variables are shown in Table 1. The gender of patients with advanced lung cancer was mostly male (73.7%), married (87%), junior high school education or below (56.6%), monthly household income of 1,000–3,000 (34.2%), primary caregiver was the patient’s spouse (50.9%), personality was introverted (41.8%), number of uncomfortable symptoms 1–3 (54.1%), and diagnosed more than one year (52.2%). The mean of DDI, CMUIS, and PGAC were 36.35 (SD = 9.25), 56.92 (SD = 15.65), and 52.29 (SD = 9.08), respectively.

**Table 1:** Sociodemographic and psychosocial characteristics (*n* = 316).

	*n*	%	*M*	SD	Range
**Gender**					
Males	233	73.7			
Females	83	26.3			
**Age**					
<60	114	36.1			
≥60	202	63.9			
**Marital status**					
Married	275	87			
Other	41	13			
**Education**					
Junior high school or less	179	56.6			
High school	123	38.9			
Bachelor and above	14	4.4			
**Household income (¥/month)**					
<1,000	27	8.5			
1,000–3,000	108	34.2			
3,000–5,000	99	31.3			
>5,000	82	25.9			
**Primary caregivers**					
Spouse	161	50.9			
Parents or Children	29	9.2			
Relatives and friends	5	1.6			
Attendant	121	38.3			
**Character**					
Introverted type	132	41.8			
Extroverted type	116	36.7			
Mixed type	68	21.5			
**Discomfort symptoms**					
0	86	27.2			
1–3	171	54.1			
>3	59	18.7			
**Diagnosis time (months)**					
<6	93	29.4			
6–12	58	18.4			
>12	165	52.2			
**MUIS Total**			56.92	15.65	27–91
Ambiguity			21.11	6.34	10–35
Lack of clarification			18.16	5.39	8–32
Unpredictability			17.65	4.86	8–25
**DDI total**			36.35	9.25	19–51
**PGAC total**			52.29	9.08	34–71
Self-awareness			24.97	4.87	15–34
Disease adjustment			7.56	1.71	4–11
Sad			7.75	1.64	4–12
Anger			6.13	1.11	3–9
Religious comfort			5.87	1.46	3–9

*M*, mean; SD, standard deviation; MUIS, Mishel uncertainty in illness scale; DDI, distress disclosure index; PGAC, preparatory grief in advanced cancer patients.

### Self-disclosure, illness uncertainty, and anticipatory grief, according to sample characteristics

As shown in Table 2, patients aged <60 reported significantly higher PDAG scores than survivors aged ≥60 (*F* = 4.541, *p* < 0.05). In terms of marital status, married patients with advanced lung cancer reported lower PDAG scores than others (*F* = 0.941, *p* < 0.01). In terms of educational attainment, patients with junior high school and below had higher scores than those with high school and college and bachelor’s degree and above (*F* = 47.206, *p* < 0.001). In terms of monthly household income, higher than 5,000 reported significantly lower scores than those of patients with 5,000 (*F* = 89.898, *p* < 0.001). In terms of primary caregiver, patients who were cared for by their spouse reported lower PDAG scores compared to those who were cared for by their parents and children or others (*F* = 2.683, *p* < 0.05). Introversion scores were higher than extroversion and mixed (*F* = 97.243, *p* < 0.001). Patients with more symptoms of discomfort had higher scores (*F* = 51.272, *p* < 0.001).

**Table 2:** AG, according to sociodemographic characteristics (*n* = 316).

Outcomes	PGAC
Gender	
Males	51.77 ± 8.86
Females	53.73 ± 9.57
*F*	1.149
*p*	0.091
Age	
<60	53.73 ± 8.37
≥60	51.48 ± 9.37
*F*	5.949
*p*	0.034*
Marital status	
Married	51.68 ± 8.99
Other	56.39 ± 8.64
*F*	0.941
*p*	0.002**
Education	
Junior high school or less	56.10 ± 8.53
High school	47.21 ± 7.04
Bachelor and above	48.14 ± 8.41
*F*	47.206
*p*	0.000***
Household income (¥/month)	
<1,000	58.26 ± 6.89
1,000–3,000	59.37 ± 6.56
3,000–5,000	49.21 ± 8.08
>5,000	44.71 ± 4.55
*F*	89.898
*p*	0.000***
Primary caregivers	
Spouse	50.91 ± 9.08
Parents or children	54.52 ± 9.54
Relatives and friends	54.40 ± 7.77
Others	53.50 ± 8.81
*F*	2.683
*p*	0.047*
Character	
Introverted type	58.25 ± 7.45
Extroverted type	45.56 ± 5.78
mixed type	52.19 ± 8.53
*F*	97.243
*p*	0.000***
Discomfort symptoms	
0	46.20 ± 6.56
1–3	52.82 ± 8.67
>3	59.61 ± 7.30
*F*	51.272
*p*	0.000***
Diagnosis time (months)	
<6	54.33 ± 9.19
6–12	54.21 ± 9.02
>12	50.46 ± 8.69
*F*	7.282
*p*	0.001**

*p < 0.05, **p < 0.01, ***p < 0.001. AG, anticipatory grief; PGAC, preparatory grief in advanced cancer patients scale.

### The correlations between self-disclosure, illness uncertainty, and anticipatory grief

Correlation coefficients for Self-disclosure, IU, and AG are shown in Table 3. Correlations between Self-disclosure (i.e., DDI total score), IU (i.e., CMUIS total score), and AG (i.e., PGAC total score) were correlated and statistically significant (*p* < 0.01). AG was negatively correlated with self-disclosure (*r* = −0.72, *p* < 0.01), and positively correlated with IU (*r* = 0.77, *p* < 0.01). Self-disclosure was negatively correlated with IU (*r* = −0.68, *p* < 0.01).

**Table 3:** Correlations (*r*) between Self-disclosure, IU, and AG (*n* = 316).

	1	2	3	4	5	6	7	8	9	10	11
1. MUIS total	1										
2. Ambiguity	0.963**	1									
3. Lack of clarification	0.939**	0.861**	1								
4. Unpredictability	0.924**	0.842**	0.792**	1							
5. DDI total	−0.684**	−0.687**	−0.620**	−0.619**	1						
6. PGAC total	0.766**	0.734**	0.684**	0.750**	−0.717**	1					
7. Self-awareness	0.751**	0.714**	0.668**	0.748**	−0.677**	0.957**	1				
8. Disease adjustment	0.599**	0.580**	0.554**	0.560**	−0.646**	0.783**	0.670**	1			
9. Sad	0.615**	0.611**	0.543**	0.583**	−0.583**	0.811**	0.711**	0.562**	1		
10. Anger	0.248**	0.229**	0.204**	0.272**	−0.270**	0.476**	0.368**	0.296**	0.305**	1	
11. Religious comfort	0.671**	0.640**	0.608**	0.651**	−0.582**	0.831**	0.751**	0.602**	0.656**	0.283**	1

**p < 0.01. IU, illness uncertainty; AG, anticipatory grief; DDI, distress disclosure index; MUIS, mishel uncertainty in illness scale; PGAC, preparatory grief in advanced cancer patients scale.

### Common method variance

The Harman one-way test was chosen to test the bias of the 58 test factors in this study, and it was found that the eigenvalues of 10 factors were > 1, and the explained variance of the first principal factor was 37.14, which was smaller than the 40% critical criterion for the existence of common method bias effect, and it was excluded that there was a common method bias effect among the 58 detection factors in this study (Podsakoff et al., 2003).

### Test of the model

**Figure 2:**
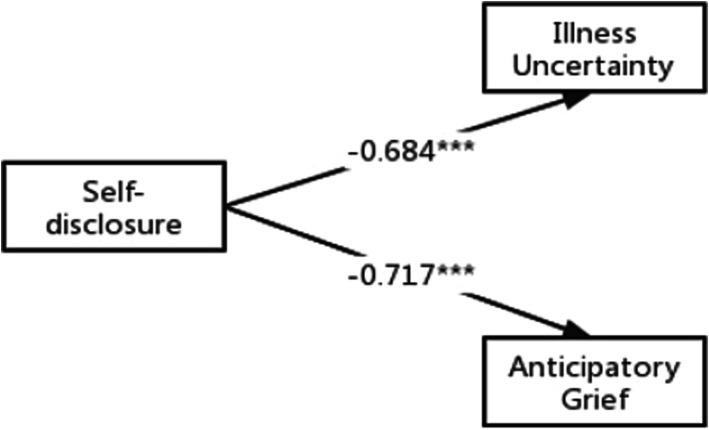
Direct effects of self-disclosure on anticipatory grief and illness uncertainty. ***p < 0.001 (two-tailed); *n* = 316.

First we tested the first mediating condition hypothesis 1. As shown in Table 3, self-disclosure can be positively correlated with predicting AG (*r* = −0.72 < 0.01). In addition, the result of the direct effect of self-disclosure on AG (standardised direct effect = −0.72 *p* < 0.001, see Figure 2) was statistically significant.

**Figure 3:**
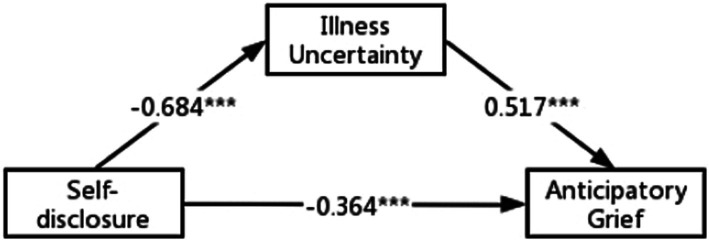
Structural equation modeling of the hypothesized model. ***p < 0.001 (two-tailed); *n* = 316.

We measured the second condition of mediation to test hypothesis 2. Correlation analyses at Table 3 indicated that self-disclosure was significantly negatively correlated with IU (*r* = −0.68, *p* < 0.01) and IU were significantly positively correlated with AG (*r* = 0.77, *p* < 0.01). In addition, the results were statistically significant for the direct effect of self-disclosure on IU (standardised direct effect = −0.69, *p* < 0.001) and for the direct effect of feelings of IU on AG (standardised direct effect = 0.52, *p* < 0.001, see Figure 3). In order to investigate the indirect effect of the dependent variable through mediation, we used Bootstrap tests (5,000 samples) to verify that the direct effect of self-disclosure on AG as well as the mediating effect of IU were statistically significant at 95% confidence intervals excluding 0 (*p* < 0.001) (Table 4). We confirms that there is a statistically significant mediating effect of IU between self-disclosure and AG (standardised indirect effect = −0.35, *p* < 0.001). The mediating effect can be evaluated by the explanatory variance ratio (VAF), VAF = indirect path coefficient/total effect path coefficient X 100%, with VAF <20% representing no mediating effect, 20–80% representing a partial mediating effect, and > 80% representing a fully mediating effect (Peirong et al., 2023). The value of VAF in this study is 49%, which indicates a partial mediated effect. Therefore, hypothesis 2 is supported.

**Table 4:** Standardized direct, indirect, and total effects of the hypothesized model.

	Point estimate	Product of coefficients		Bootstrapping				
				Bias-corrected 95% CI		Percentile 95% CI		Two-tailed significance
		SE	*Z*	Lower	Upper	Lower	Upper	
Direct effects								
SD → AG	−0.364	0.047	−7.745	−0.457	−0.270	−0.457	−0.270	0.001**
Indirect effects								
SD → AG	−0.353	0.034	−10.282	−0.422	−0.291	−0.422	−0.291	0.001**
Total effects								
SD → AG	−0.717	0.024	−29.875	−0.760	−0.667	−0.760	−0.667	0.001**

Estimating of 5,000 bootstrap sample, ***p < 0.001. SD, self-disclosure; AG, anticipatory grief.

## Discussion

The purpose of this study was to investigate the correlation between self-disclosure and IU and AG in Chinese patients with advanced lung cancer. To the best of our knowledge, this study is the first to examine the relationship between the three in a sample of Chinese advanced lung cancer patients. As hypothesised, we observed that patients’ AG levels were at a medium-high level, and we also found support for the hypothesis that lower AG is associated with higher self-disclosure. Finally, IU in patients with advanced lung cancer partially mediated this relationship.

AG is a common emotional response in advanced cancer patients, and the score of this study was 52.29 + 9.08. Although there no cut-off, AG does not require a diagnosis and can be psychologically interventional (Davis et al., 2017). As shown in Table 2 advanced lung cancer patients aged <60 are more likely to suffer from high levels of AG, it may be that patients at a lower age need to take on more family responsibilities, and the conflict between the energy they need to give and the energy they are able to give is one of the reasons for exacerbating AG (Zhengwei et al., 2022). In contrast, patients aged ≥60 years their role in the family may be more to take care of themselves without sharing too much responsibility for the family, and there is more life experience to help them cope better with AG. Lower levels of education tend to have higher AG scores, cancer itself is a more complex being with greater individual variability, and it is a difficult process for patients with low levels of education to go from a nascent desire to learn about the disease to being able to familiarise themselves with the disease, which is consistent with previous finding in 235 patients with nasopharyngeal cancer (Xian and Lin, 2022). In line with the finding of 301 patients with liver cancer, AG levels would be lower in patients who are married with a sizable income and have a spouse to care for them, possibly because the long-term companionship of the spouse enhances the patient’s intimate relationship with the caregiver, which is an important influencing factor for self-disclosure (Du et al., 2021; Chunyan et al., 2023). On the one hand, patients with advanced lung cancer mainly rely on basic medical insurance to pay for their medical expenses, and the limited variety of anti-cancer drugs currently covered by medical insurance has resulted in patients spending more on anti-cancer treatment than they can afford. On the other hand, cancer treatment creates both psychological and physical barriers that lead to temporary or permanent unemployment. The financial problems of patients with low family income may become the primary stressor and they are always faced with the inability to pay forcing them to stop or voluntarily give up the treatment, which leads to high levels of AG recurring. Therefore, social security services should take care of low-income or unemployed groups and provide them with employment or financial assistance whenever possible. Introverts tend to have higher AG scores, most introverts are not very expressive and with their own illnesses they may prefer to choose to keep themselves to themselves. In contrast, extroverts are willing to talk to others and like to express their thoughts, and in the exposure emotional cognition might change thereby lowering AG (Zhu et al., 2023). Symptoms affect the patient’s view of the disease, causing biased perceptions (Guan et al., 2020). In the study, the IU for patients with advanced lung cancer was 56.92 ± 15.65, which is in the middle to high range. Probably due to the lack of knowledge of the disease in advanced cancer patients, when one or more physical discomfort symptoms (such as pain, sleep disorder, loss of appetite, etc.) arise in the process of receiving treatment, they tend to feel blind and helpless, and produce many doubts while guessing about the disease (often in a bad way), but they do not want to or dare not to express it, probably fearing to get a definite answer about the deterioration of the disease, thus This creates ambivalence and constant internal conflict, which leads to further negative emotions such as anxiety and grief (Sasai and Onishi, 2017), and negative coping styles such as avoidance and passivity, thus greatly reducing adherence to cancer treatment and quality of life in late stages (Han et al., 2021). Finally, in this study, it was found that the self-disclosure score of advanced lung cancer patients was 36.35 ± 9.25, which was at a moderate levels indicates that there is much room for improvement in the self-disclosure of Chinese cancer patients. On the one hand, because cancer is extremely traumatic to individuals and families, patients tend to develop a heavier burden of self-feelings (Han et al., 2021), leading to changes in the patient’s personality, when in order to reduce the pressure on the family, they choose to bear the psychological distress caused by the losses due to the development of the disease alone, and if the family members or healthcare professionals do not detect and give correct guidance in time, the patient will develop serious psychological problems. On the other hand, for lung cancer patients, smoking is often perceived as uncivilised, thus increasing the patients’ sense of shame about their illness, leading them to choose to remain closed and gradually lose the ability to share. As the disease progresses and the physical condition changes, patients are surrounded by uncertainty, confusion, and unknowns, which naturally lead to the formation of AG (Sun et al., 2022). If healthcare professionals do not provide proper intervention and guidance at an early stage, patients will develop serious psychological problems.

This study found a negative direct effect between self-disclosure and AG, which is line with our proposed hypothesis 1. Suggesting that advanced lung cancer patients with higher self-disclosure experience lower levels of AG. Positive self-disclosure promotes adjustment and adaptation to stressors, alleviates patients’ negative psychological experiences, and reduces AG levels in cancer patients. The reason is that self-disclosure is a positive emotion regulation method. Patients with advanced lung cancer can clarify their needs by talking with family and friends, and obtain emotional recognition and support. Self-disclosure can change patients’ cognitive evaluation. By telling their views and attitudes about what happened before, is experiencing, or will happen in the future, the listener (caregiver or medical staff) can give positive responses and provide professional answers to patients’ confusion to relieve their doubts, so that patients can re-evaluate the negative thoughts caused by cancer, reduce intrusive thoughts and hypervigilance. This results in a positive cognitive evaluation and reduced IU and AG levels (Zhou et al., 2021). For advanced lung cancer patients with low self-disclosure level, medical staff should gain the trust of patients, create a comfortable self-disclosure environment for them, encourage them to communicate more with family, friends and medical staff, and improve their willingness to self-disclosure.

The results of this study confirm our second hypothesis that IU plays a partially mediating role in the negative effect of self-disclosure on AG. This shows that when patients with advanced lung cancer face stressful events and insufficient self-disclosure, they will produce IU, live in IU for a long time, but cannot make correct cognitive evaluation, so that patients will face great psychological pressure, leading to negative development of AG (Li et al., 2022). This illustrates the importance of IU in the development of AG, and the perception and understanding of stressful disease events in cancer patients is often cited as a key factor in the incidence of IU (Zhang, 2017). Self-disclosure enables cancer patients to express their cognitive views and emotional attitudes, give others more opportunities to understand them and provide more and better help, improve patients’ subjective perception and self-coping skills, and reduce the stress disease events assessed by IU severity. Therefore, interventions to alleviate IU and AG in patients with advanced lung cancer may be to improve the level of self-disclosure of patients, increase doctor-patient communication and promote the exchange of disease-related information, requiring healthcare professionals to create an open and free disclosure environment, guide patients to express their emotions when they feel sad and think about the future with family and friends.

### Limitations

This study has the following limitations: firstly, this study is a cross-sectional study, and it is not possible to explore the causal relationship between the study variables. Secondly, this study was single-center and the convenience sampling method was used to limit the diversity of results, so the results may not represent the total number of patients diagnosed with advanced lung cancer. Future multi-center randomized experiments should be conducted. In addition, although the scales used in this study were all of high reliability, the self-reported collection of survey variables may result in reporting bias, and multiple assessments or improved experimental designs should be conducted in the future to reduce the degree of bias or avoid bias. Finally, this study only considered the effect of self-disclosure on AG and the mediating effect of IU between the two. In the future, we will consider more influencing variables, and we can improve the analysis of the influencing factors by adding objective indicators (such as immune indicators, cortisol, etc.) such as laboratory tests, so as to provide the basis for the development of more targeted and practical interventions.

### Implications

This study has the following implications for the understanding and intervention of anticipatory grief. First, self-disclosure has a great impact on anticipatory grief in Chinese advanced lung cancer patients, suggesting that improving the level of self-disclosure will help reduce anticipatory grief. Second, patients with advanced lung cancer appear to ease anticipatory grief by reducing uncertainty about the disease. For example, to encourage patients to express their inner feelings and doubts, targeted measures can be developed to improve the level of self-disclosure of patients with advanced lung cancer, and medical staff can provide professional answers to give patients more emotional comfort and reliable answers, thereby reducing their level of anticipatory grief.

## Conclusion

AG in advanced lung cancer patients is at an intermediate to high level. There is a correlation between Self-disclosure, IU and AG, with IU playing a partial mediating role between Self-disclosure and AG. To maintain AG in advanced lung cancer patients within a healthy range, healthcare staff should pay attention to and evaluate the level of AG in advanced lung cancer patients, encourage patients to express their emotions and suspicions to others, and healthcare staff as well as patients’ family members should respond positively to form a positive circle, which is conducive to improving patients’ ability of self-disclosure as well as decreasing the IU, and diminishing the adverse effects of AG.

## Data availability statement

The raw data supporting the conclusions of this article will be made available by the authors, without undue reservation.

## Ethics statement

The studies involving humans were approved by Ethics Committee of Jiangnan University. The studies were conducted in accordance with the local legislation and institutional requirements. The participants provided their written informed consent to participate in this study. Written informed consent was obtained from the individual(s) for the publication of any potentially identifiable images or data included in this article.

## Author contributions

NZ: Investigation, Methodology, Writing – original draft, Writing – review & editing. HL: Investigation, Writing – original draft. HK: Investigation, Writing – original draft. YW: Investigation, Writing – original draft. ZZ: Data curation, Investigation, Methodology, Writing – review & editing.
